# Mental Multimorbidity Among General-Population Adults: Sex-Specific Sociodemographic Profiles of Anxiety, Insomnia, and Eating Disorders

**DOI:** 10.3389/ijph.2024.1607546

**Published:** 2024-10-28

**Authors:** Valentina A. Andreeva, Nathalie Arnault, Stéphanie Chambaron, Cécilia Samieri, Marie-Claude Brindisi, Pauline Duquenne, Serge Hercberg, Pilar Galan, Mathilde Touvier, Leopold K. Fezeu

**Affiliations:** ^1^ Nutritional Epidemiology Research Group, Sorbonne Paris Nord University and University of Paris, INSERM/INRAE/CNAM, Epidemiology and Statistics Research Center, Bobigny, France; ^2^ Center for Taste and Feeding Behavior, CNRS/INRAE/Agro Institute, University of Bourgogne, Dijon, France; ^3^ University of Bordeaux, INSERM, Bordeaux Population Health Research Center UMR1219, Bordeaux, France

**Keywords:** anxiety, eating disorders, insomnia, mental multimorbidity, general population

## Abstract

**Objective:**

To determine the prevalence and sociodemographic profiles of mental morbidity and multimorbidity.

**Methods:**

A descriptive analysis was performed with data from 25,269 women and 8,389 men from the French NutriNet-Santé general-population cohort. Participants were split into 8 groups: 1. No mental morbidity; 2. Pure anxiety; 3. Pure insomnia; 4. Pure eating disorders (ED); 5. Comorbid anxiety and insomnia; 6. Comorbid anxiety and ED; 7. Comorbid insomnia and ED; 8. Multimorbid anxiety, insomnia, and ED. Data were weighted using the 2016 French Census and analyzed with Chi^2^ tests.

**Results:**

40.6% of the participants had ≥1 mental disorder; 2.3% had all 3 disorders. Most pure and comorbid disorders were more common in women than in men. The multimorbidity group had the largest proportions of men who were overweight (52.1%) and current smokers (23.2%). Men with insomnia and ED were the most likely to have obesity (45.8%) and low physical activity (44.3%). Women with ≥2 disorders were the most likely to be current smokers.

**Conclusion:**

The findings could inform research, prevention, and public health guidelines for multimorbidity.

## Introduction

Suffering from ≥2 chronic conditions is termed comorbidity when one of the conditions is regarded as primary or multimorbidity (without a primary condition) [1]. Such phenomena are associated with symptom severity, increased healthcare utilization, and a poorer prognosis [2, 3]. The vast majority of comorbidity/multimorbidity research understandably addresses physical illnesses in elderly populations [4, 5]. From a public health viewpoint and irrespective of age, mental health receives less attention than physical health, despite its substantial contribution to disease burden, further worsened by the COVID-19 pandemic [6] and the marked reduction in life expectancy among individuals with mental illness compared with the general population [7, 8]. Findings from the 27-country World Health Organization (WHO) Mental Health Surveys revealed that each lifetime mental disorder was associated with a substantially increased risk of subsequent mental comorbidity, with the risk persisting over >15 years [9].

Here we focus on three mental health conditions - anxiety, insomnia, and eating disorders (ED) - because they are relatively frequent in the general population, display a strong potential for comorbidity, and offer opportunities for prevention or treatment [10–12]. The most common among them is anxiety (encompassing generalized anxiety disorder, panic disorder, social anxiety, phobias, etc.) with prevalence estimates of 4%–25%, with higher rates among women, younger adults, and individuals with chronic illness [13]. During 1990–2019, disability-adjusted life years attributable to anxiety disorders increased worldwide by nearly 54% across age [14]. Next, a meta-analysis reported that not only anxiety but also many other mental disorders were concomitant with sleep disturbances, implying an imbalance in the arousal system [15]. Moreover, network analyses have provided evidence for connections among symptoms of anxiety, sleep disorders and ED [11].

The U.S. National Comorbidity Survey (baseline: 1990–1992; replication: 2001–2003) was among the first to address the prevalence and correlates of comorbid and multimorbid mental disorders [3, 16]. However, to date, few epidemiological studies have investigated the socio-demographic profiles of pure versus comorbid/multimorbid mental disorders in the general population [17–20] which is indispensable for identifying at-risk subgroups. To our knowledge, no prior epidemiological research has addressed the multimorbidity of anxiety, sleep disturbance/insomnia and ED in general-population adults. Only one small cross-sectional study with 130 Brazilian adults with overweight or obesity included measures of all three disorders and reported that adults aged <45 years with high trait anxiety also had high scores on binge eating and low scores on sleep quality, based on two-way correlations [21].

There is compelling need to advance mental multimorbidity research to inform targeted public health interventions. Therefore, this descriptive study investigated the prevalence and degree of mental multimorbidity, focusing on three mental health conditions and the socio-demographic characteristics of pure versus multimorbid cases. Given that women present higher rates of anxiety [13], insomnia [22] and ED [23], we explored sex-specific associations.

## Methods

### Research Context

This analysis is part of the 4-year MEMORIES Project, launched in France in 2022 (https://memories-anr.univ-paris13.fr/) and aimed at elucidating the risk of developing metabolic disorders (obesity and type 2 diabetes) associated with mental morbidity and multimorbidity [24].

### Study Population

Epidemiological data for MEMORIES came from the ongoing NutriNet-Santé e-cohort (https://etude-nutrinet-sante.fr/) launched in 2009. Its design and objectives are detailed elsewhere [25]. Briefly, adults aged ≥18 years who comprehend written French and are able to follow an online protocol are recruited from the general population via media campaigns. NutriNet-Santé was approved by the Institutional Review Board of the French Institute for Health and Medical Research and by the National Commission on Informatics and Liberty. Electronic informed consent is obtained from each volunteer prior to enrollment.

Data are collected via self-report questionnaires. Sociodemographic, anthropometric, lifestyle, diet, physical activity, and health status information is gathered at inclusion and annually thereafter. Over the follow-up, participants complete additional questionnaires on nutrition or health-related topics on a voluntary basis. All mental health assessments (described below) took place as part of the general follow-up of the cohort.

### Anxiety Assessment

The 20-item trait anxiety subscale of the State-Trait Anxiety Inventory Form Y (STAI-T) was used for evaluating general anxiety proneness, distinguishing it from depression [26, 27]. Trait anxiety measured by STAI-T was reported to be highly correlated with generalized anxiety disorder [28]. Each item is scored on a 4-point Likert scale ranging from “Almost never” to “Almost always.” The higher the score, the greater the proneness to anxiety. STAI-T was administered during 2013–2016 and each participant completed it only once. Of the 119,451 enrollees solicited, n = 40,809 responded. As in prior epidemiological research [29], we modelled STAI-T in quartiles (Q), with Q4 as the sex-specific cutoff defining high trait anxiety (men Q4 ≥ 41; women Q4 ≥ 46).

### Insomnia Assessment

A sleep questionnaire was administered in 2014; of the 128,042 solicited participants, n = 57,105 responded within 6 months. The questionnaire included items about sleep duration, chronotype, acute and chronic insomnia, napping, and stress-related sleep disturbance. Here we focused on chronic insomnia assessed according to the criteria established by the Diagnostic and Statistical Manual of Mental Disorders - 5th Edition (DSM-5) [30] and the International Classification of Sleep Disorders - 3rd Edition (ICSD-3) [31]. These criteria were included as questionnaire items and provide information about difficulties falling asleep and/or frequent nighttime wakening ≥3 nights/week, over the past ≥3 months, and the experience of negative repercussions of such problems in daily life.

### Eating Disorder (ED) Assessment

We screened for any ED, without distinguishing the type, in 2014 and 2017 using the 5-item SCOFF questionnaire [32, 33]. Each item (e.g., “Do you worry you have lost control over how much you eat?; “Would you say food dominates your life?) is dichotomous (Yes/No); an ED threshold is fixed at ≥2 affirmative responses, with sensitivity and specificity >94% using interviews as diagnostic reference [33]. Data obtained with SCOFF are regarded as reflecting likely ED, approximating actual ED point prevalence [34]. Of the 125,279 enrollees who received SCOFF in 2014, a total of n = 51,073 responded within 6 months. Participants who did not complete SCOFF in 2014 but did so in 2017 (n = 6,570) were also eligible for the study.

### Sociodemographic Profile Assessment

At inclusion and annually thereafter, data on sex, age, marital status, education, occupation, smoking, anthropometrics, and physical activity were collected from all participants, using validated socio-demographic [35] and anthropometric [36, 37] questionnaires. From the reported weight and height, the body mass index (BMI, kg/m^2^) of each participant was calculated. Next, physical activity was assessed with the short form of the International Physical Activity Questionnaire (IPAQ) and scoring followed an established algorithm [38].

For each participant, we first calculated the average date of completion of the three mental health assessments which covered the period 2013–2017. Next, we selected covariable data obtained within a 2-year window around that average date. Individuals with missing data on any covariables were ineligible for analysis.

### Statistical Analysis

Scores on each of the three mental health measures were dichotomized for each participant to reflect the presence or absence of the respective condition; 8 morbidity groups were thus created: no mental morbidity, pure anxiety, pure insomnia, pure ED (any type), comorbid anxiety and insomnia, comorbid anxiety and ED, comorbid insomnia and ED, multimorbid anxiety, insomnia, and ED. Next, we investigated the prevalence of mental morbidity and the sociodemographic characteristics of participants with one, two and all three mental health conditions. The following characteristics served as exposures: age (as a continuous and a 3-level variable: 18–39, 40–59, ≥60 years), marital status (living alone, married/cohabiting), education (< high school, high school or equivalent, some college, undergraduate/graduate degree), occupation/employment (without professional activity, self-employed/artisan/farmer, blue-collar worker, office/administrative staff, professional/executive staff, retired), BMI [kg/m^2^, as a continuous and a 4-level variable: underweight (<18.5), normal weight (18.5–24.9), overweight (25.0–29.9), obesity (≥30.0)], smoking status (never, former, current smoker), and physical activity level (low, moderate, high).

Statistical calibration was applied to the data via the SAS CALMAR macro developed by the French Census Bureau [39]. Specifically, we calculated and applied statistical weights according to the sex-, age- and socioeconomic status distribution in the 2016 French Census. The weighted data were then analyzed with Chi^2^ tests using SAS version 9.4 (SAS Institute, Inc., Cary NC, United States).

## Results

**FIGURE 1:**
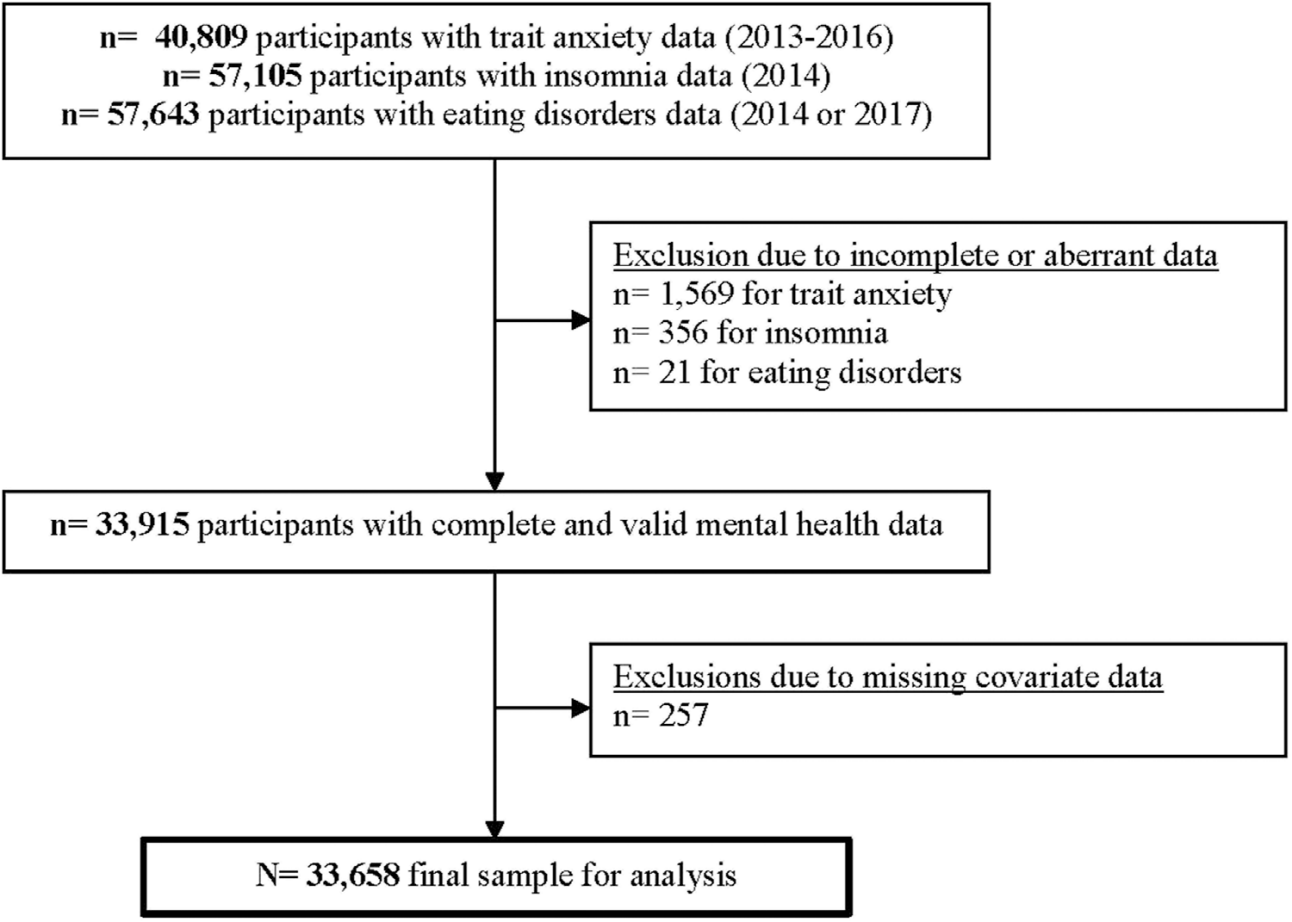
NutriNet-Santé participant selection flowchart (France, 2013–2017).

In total, N = 33,658 participants (25,269 women, 8,389 men) had complete and valid mental health and covariate data and were included in the analysis (Figure 1). The overall and sex-specific raw and weighted distribution of mental morbidity prevalence is summarized in Table 1. Overall, 40.6% (men 37.4%; women 43.6%) had ≥1 mental health condition; 2.3% had all three conditions (men 1.4%; women 3.1%). Across sex, the most prevalent comorbidity was anxiety and insomnia (men 6.1%; women 6.4%) whereas the least prevalent was insomnia and ED (men 0.9%; women 1.6%). The raw and weighted sociodemographic profiles associated with mental morbidity are presented in Table 2 (women) and Table 3 (men).

**TABLE 1:** Distribution of mental morbidity prevalence: anxiety, insomnia, and eating disorders (NutriNet-Santé cohort, France. 2013–2017).

	Full sample N = 33,658			Men n = 8,389			Women n = 25,269			Chi-squared test *p*-value
	Raw data No. (%)		*W* ^a^ *%*	Raw data No. (%)		*W* *%*	Raw data No. (%)		*W* *%*	
General (trait) anxiety^b^										0.42
No	25,056	(74.4)	*74.6*	6,273	(74.8)	*74.1*	18,783	(74.3)	*75.0*	
Yes	8,602	(25.6)	*25.4*	2,116	(25.2)	*25.9*	6,486	(25.7)	*25.0*	
Chronic insomnia^c^										<0.0001
No	26,933	(80.0)	*81.5*	7,274	(86.7)	*85.2*	19,659	(77.8)	*78.2*	
Yes	6,725	(20.0)	*18.5*	1,115	(13.3)	*14.8*	5,610	(22.2)	*21.8*	
Eating disorder (any type)^d^										<0.0001
No	29,376	(87.3)	*87.9*	7,757	(92.5)	*91.0*	21,619	(85.6)	*85.1*	
Yes	4,282	(12.7)	*12.1*	632	(7.5)	*9.0*	3,650	(14.4)	*14.9*	
Degree of mental multimorbidity										<0.0001
None	19,508	(58.0)	*59.4*	5,413	(64.5)	*62.6*	14,095	(55.8)	*56.4*	
General (trait) anxiety	4,453	(13.2)	*13.6*	1,368	(16.3)	*15.8*	3,085	(12.2)	*11.7*	
Chronic insomnia	3,235	(9.6)	*8.7*	508	(6.1)	*6.5*	2,727	(10.8)	*10.6*	
Eating disorder only (any type)	1,824	(5.4)	*5.2*	300	(3.6)	*4.1*	1,524	(6.0)	*6.3*	
Anxiety and insomnia	2,180	(6.5)	*6.3*	468	(5.6)	*6.1*	1,712	(6.8)	*6.4*	
Anxiety and eating disorders	1,148	(3.4)	*3.2*	193	(2.3)	*2.6*	955	(3.8)	*3.8*	
Insomnia and eating disorders	489	(1.5)	*1.3*	52	(0.6)	*0.9*	437	(1.7)	*1.6*	
Anxiety, insomnia, and eating disorders	821	(2.4)	*2.3*	87	(1.0)	*1.4*	734	(2.9)	*3.1*	

^a^ W = percentage obtained by applying statistical weights according to sex-, age- and socioeconomic status distribution in the 2016 French Census.

^b^ General anxiety proneness evaluated with STAI-T, applying sex-specific 75% cutoffs (men: 41 points; women: 46 points).

^c^ Chronic insomnia defined according to DSM-5 and ICSD-3 criteria.

^d^ Presence of any eating disorder defined as ≥2 affirmative responses on the SCOFF questionnaire.

**TABLE 2:** Socio-demographic characteristics of women according to mental morbidity status (NutriNet-Santé, n = 25,269, France. 2013–2017).

	No mental morbidity (n = 14,095)		Pure anxiety^a^ (n = 3,085)		Pure insomnia^b^ (n = 2,727)		Pure eating disorders^c^ (n = 1,524)		Anxiety and insomnia (n = 1,712)		Anxiety and eating disorders (n = 955)		Insomnia and eating disorders (n = 437)		Anxiety, insomnia, and eating disorders (n = 734)	
	raw data No. (%)	*W* ^d^ *%*	raw data No. (%)	*W* *%*	raw data No. (%)	*W* *%*	raw data No. (%)	*W* *%*	raw data No. (%)	*W* *%*	raw data No. (%)	*W* *%*	raw data No. (%)	*W* *%*	raw data No. (%)	*W* *%*
Age, *years*, mean, SD	51.1 (13.9)		48.3 (14.0)		52.7 (12.1)		49.3 (13.8)		51.0 (12.6)		46.4 (14.0)		51.9 (11.6)		47.9 (12.7)	
Age categories																
18–39 *years*	3,521 (25.0)	*30.4*	985 (31.9)	*39.2*	464 (17.0)	*20.3*	423 (27.8)	*39.5*	367 (21.5)	*30.6*	344 (36.0)	*45.9*	72 (16.5)	*19.9*	211 (28.8)	*37.3*
40–59 *years*	5,825 (41.3)	*40.2*	1,282 (41.6)	*40.7*	1,396 (51.2)	*52.8*	654 (42.9)	*42.7*	865 (50.5)	*52.2*	392 (41.1)	*42.1*	237 (54.2)	*58.9*	376 (51.2)	*52.2*
60+ *years*	4,749 (33.7)	*29.4*	818 (26.5)	*20.1*	867 (31.8)	*26.9*	447 (29.3)	*17.8*	480 (28.0)	*17.2*	219 (22.9)	*12.0*	128 (29.3)	*21.2*	147 (20.0)	*10.5*
Marital status																
Living alone	3,531 (25.0)	*27.5*	884 (28.6)	*30.6*	674 (24.7)	*22.8*	403 (26.4)	*28.2*	501 (29.3)	*28.4*	299 (31.3)	*34.1*	116 (26.5)	*28.2*	240 (32.7)	*33.4*
Married, cohabiting	10,564 (75.0)	*72.5*	2,201 (71.4)	*69.4*	2,053 (75.3)	*77.2*	1,121 (73.6)	*71.8*	1,211 (70.7)	*71.6*	656 (68.7)	*65.9*	321 (73.5)	*71.8*	494 (67.3)	*66.6*
Educational level																
<High school	2,315 (16.4)	*19.2*	527 (17.1)	*21.3*	432 (15.8)	*19.7*	314 (20.6)	*21.4*	320 (18.7)	*21.4*	173 (18.1)	*19.4*	92 (21.0)	*27.5*	153 (20.8)	*24.9*
High school or equivalent	1,854 (13.2)	*15.8*	458 (14.8)	*16.8*	375 (13.8)	*15.6*	214 (14.0)	*16.6*	283 (16.5)	*20.2*	153 (16.0)	*22.2*	71 (16.3)	*21.8*	124 (16.9)	*20.6*
Some college	4,796 (34.0)	*33.4*	954 (30.9)	*29.1*	1,001 (36.7)	*32.0*	519 (34.1)	*32.2*	582 (34.0)	*31.1*	337 (35.3)	*30.4*	145 (33.2)	*29.0*	254 (34.6)	*30.1*
Undergraduate/graduate	5,130 (36.4)	*31.6*	1,146 (37.2)	*32.8*	919 (33.7)	*32.7*	477 (31.3)	*29.8*	527 (30.8)	*27.3*	292 (30.6)	*28.0*	129 (29.5)	*21.7*	203 (27.7)	*24.4*
Occupation/employment																
No professional activity^e^	1,409 (10.0)	*28.8*	458 (14.8)	*35.1*	313 (11.5)	*29.6*	148 (9.7)	*22.9*	246 (14.4)	*31.7*	151 (15.8)	*33.6*	60 (13.7)	*31.2*	137 (18.7)	*36.5*
Self-employed, artisan	283 (2.0)	*2.5*	38 (1.2)	*1.4*	65 (2.4)	*3.1*	30 (2.0)	*2.5*	34 (2.0)	*2.6*	22 (2.3)	*2.8*	7 (1.6)	*1.9*	11 (1.5)	*1.9*
Blue-collar worker	2,116 (15.0)	*26.7*	597 (19.4)	*30.5*	413 (15.2)	*26.8*	310 (20.3)	*38.2*	351 (20.5)	*34.3*	230 (24.1)	*37.2*	83 (19.0)	*31.5*	166 (22.6)	*36.8*
Administrative staff	2,425 (17.2)	*7.9*	531 (17.2)	*7.2*	492 (18.0)	*8.5*	244 (16.0)	*7.8*	293 (17.1)	*7.3*	161 (16.9)	*6.5*	88 (20.2)	*9.9*	126 (17.2)	*6.7*
Executive staff	3,316 (23.5)	*16.5*	690 (22.4)	*14.2*	603 (22.1)	*15.4*	350 (23.0)	*15.6*	341 (19.9)	*12.9*	190 (19.9)	*12.8*	87 (19.9)	*14.3*	144 (19.6)	*11.7*
Retired	4,546 (32.3)	*17.6*	771 (25.0)	*11.6*	841 (30.8)	*16.6*	442 (29.0)	*13.0*	447 (26.1)	*11.2*	201 (21.0)	*7.1*	112 (25.6)	*11.2*	150 (20.4)	*6.4*
BMI, *kg/m* ^2^, mean, SD	23.4 (4.1)		23.2 (4.2)		23.8 (4.4)		25.8 (5.7)		23.6 (4.9)		25.3 (6.4)		26.4 (5.7)		25.9 (6.4)	
BMI categories																
Underweight <18.5	736 (5.2)	*6.1*	209 (6.8)	*7.4*	139 (5.1)	*6.3*	56 (3.7)	*4.4*	128 (7.5)	*8.8*	83 (8.7)	*10.1*	9 (2.1)	*3.3*	56 (7.3)	*9.1*
Normal weight 18.5–24.9	9,689 (68.7)	*67.0*	2,081 (67.5)	*65.0*	1,755 (64.4)	*65.1*	755 (49.5)	*51.0*	1,080 (63.1)	*60.4*	456 (47.8)	*50.3*	193 (44.2)	*41.7*	331 (45.1)	*46.9*
Overweight 25.0–29.9	2,678 (19.0)	*20.1*	587 (19.0)	*21.3*	584 (21.4)	*19.6*	393 (25.8)	*25.4*	330 (19.3)	*18.8*	232 (24.3)	*22.4*	135 (30.9)	*32.0*	168 (22.9)	*21.5*
Obesity ≥30.0	992 (7.1)	*6.8*	208 (6.7)	*6.3*	249 (9.1)	*9.0*	320 (21.0)	*19.2*	174 (10.1)	*12.0*	184 (19.2)	*17.2*	100 (22.8)	*23.0*	179 (24.7)	*22.5*
Smoking status																
Never smoker	7,704 (54.7)	*54.0*	1,712 (55.5)	*57.2*	1,381 (50.6)	*48.4*	749 (49.1)	*52.0*	872 (50.9)	*49.6*	503 (52.7)	*49.4*	179 (41.0)	*38.0*	325 (44.3)	*46.0*
Former smoker	4,951 (35.1)	*34.7*	997 (32.3)	*30.2*	1,063 (39.0)	*40.6*	603 (39.6)	*36.1*	645 (37.7)	*38.1*	313 (32.8)	*31.8*	206 (47.1)	*46.9*	297 (40.4)	*37.3*
Current smoker	1,440 (10.2)	*11.3*	376 (12.2)	*12.6*	283 (10.4)	*11.0*	172 (11.3)	*11.9*	195 (11.4)	*12.3*	139 (14.5)	*18.8*	52 (11.9)	*15.1*	112 (15.3)	*16.7*
Physical activity level^f^																
Low	3,003 (21.3)	*22.0*	941 (30.5)	*33.3*	660 (24.2)	*23.4*	372 (24.4)	*24.5*	456 (26.6)	*27.6*	283 (29.6)	*32.0*	133 (30.4)	*28.5*	238 (32.4)	*33.0*
Moderate	6,182 (43.9)	*44.3*	1,292 (41.9)	*40.7*	1,149 (42.1)	*44.3*	629 (41.3)	*42.0*	687 (40.1)	*36.9*	402 (42.1)	*36.2*	151 (34.6)	*39.1*	285 (38.8)	*37.3*
High	4,910 (34.8)	*33.7*	852 (27.6)	*26.0*	918 (33.7)	*32.3*	523 (34.3)	*33.5*	569 (33.3)	*35.5*	270 (28.3)	*31.8*	153 (35.0)	*32.4*	211 (28.8)	*29.7*

Values refer to number (percent) except when noted otherwise. Categorical variables compared across mental morbidity status using chi-squared tests; all *p* < 0.0001.

BMI, body mass index.

^a^General anxiety proneness evaluated with the STAI-T, applying sex-specific 75% cutoffs (men: 41 points; women: 46 points).

^b^Chronic insomnia defined according to DSM-5, and ICSD-3, criteria.

^c^Presence of any eating disorder defined as ≥2 affirmative responses on the SCOFF questionnaire.

^d^W = percentage obtained by applying statistical weights according to sex-, age- and socioeconomic status distribution in the 2016 French Census.

^e^The category includes individuals who are unemployed, homemakers, on sick leave, students, or interns.

^f^Physical activity was evaluated with the short form of the IPAQ, and scoring followed an established algorithm.

**TABLE 3:** Socio-demographic characteristics of men according to mental morbidity status (NutriNet-Santé, n = 8,389, France. 2013–2017).

	No mental morbidity (n = 5,413)		Pure anxiety^a^ (n = 1,368)		Pure insomnia^b^ (n = 508)		Pure eating disorders^c^ (n = 300)		Anxiety and insomnia (n = 468)		Anxiety and eating disorders (n = 193)		Insomnia and eating disorders (n = 52)		Anxiety, insomnia, and eating disorders (n = 87)	
	raw data No. (%)	*W* ^d^ *%*	raw data No. (%)	*W* *%*	raw data No. (%)	*W* *%*	raw data No. (%)	*W* *%*	raw data No. (%)	*W* *%*	raw data No. (%)	*W* *%*	raw data No. (%)	*W* *%*	raw data No. (%)	*W* *%*
Age, *years*, mean, SD	58.7 (12.9)		54.2 (14.4)		58.8 (12.2)		60.4 (11.5)		54.2 (12.6)		55.1 (13.4)		57.8 (12.3)		54.9 (11.5)	
Age categories																
18–39 *years*	584 (10.8)	*31.9*	268 (19.6)	*41.0*	41 (8.1)	*12.5*	22 (7.3)	*15.6*	58 (12.4)	*27.2*	34 (17.6)	*31.7*	6 (11.5)	*8.3*	12 (13.8)	*29.0*
40–59 *years*	1,660 (30.7)	*43.8*	513 (37.5)	*43.5*	187 (36.8)	*64.1*	88 (29.3)	*58.2*	231 (49.4)	*60.5*	70 (36.3)	*56.5*	20 (38.5)	*72.0*	44 (50.6)	*61.0*
60+ *years*	3,169 (58.5)	*24.3*	587 (42.9)	*15.5*	280 (55.1)	*23.4*	190 (63.4)	*26.2*	179 (38.4)	*12.3*	89 (46.1)	*11.8*	26 (50.0)	*19.7*	31 (35.6)	*10.0*
Marital status																
Living alone	818 (15.1)	*26.6*	323 (23.6)	*40.2*	85 (16.7)	*23.7*	40 (13.3)	*17.0*	128 (27.3)	*40.7*	54 (28.0)	*60.7*	7 (13.5)	*15.0*	17 (19.5)	*20.8*
Married, cohabiting	4,595 (84.9)	*73.4*	1,045 (76.4)	*59.8*	423 (83.3)	*76.3*	260 (86.7)	*83.0*	340 (72.7)	*59.3*	139 (72.0)	*39.3*	45 (86.5)	*85.0*	70 (80.5)	*79.2*
Educational level																
< High school	1,377 (25.4)	*24.0*	297 (21.7)	*23.6*	115 (22.7)	*30.7*	113 (37.7)	*35.6*	99 (21.1)	*30.3*	64 (33.2)	*52.9*	20 (38.5)	*60.1*	26 (29.9)	*22.8*
High school or equivalent	620 (11.5)	*15.6*	174 (12.7)	*14.1*	55 (10.8)	*13.9*	37 (12.3)	*22.7*	65 (13.9)	*12.2*	27 (14.0)	*17.2*	7 (13.5)	*6.6*	14 (16.1)	*26.1*
Some college	1,330 (24.6)	*29.4*	338 (24.7)	*25.7*	125 (24.6)	*23.3*	58 (19.3)	*20.3*	121 (25.9)	*23.8*	50 (25.9)	*15.4*	10 (19.2)	*12.7*	20 (23.0)	*26.9*
Undergraduate/graduate	2,086 (38.5)	*31.0*	559 (40.9)	*36.6*	213 (41.9)	*32.1*	92 (30.7)	*21.4*	183 (39.1)	*33.7*	52 (26.9)	*14.5*	15 (28.8)	*20.6*	27 (31.0)	*24.2*
Occupation/employment																
No professional activity^e^	155 (2.9)	*23.7*	92 (6.7)	*35.2*	20 (3.9)	*25.3*	9 (3.0)	*19.9*	35 (7.5)	*35.5*	17 (8.8)	*41.9*	3 (5.8)	*28.6*	8 (9.2)	*39.2*
Self-employed, artisan	109 (2.0)	*7.9*	30 (2.2)	*4.4*	10 (2.0)	*6.3*	3 (1.0)	*2.1*	19 (4.1)	*8.1*	2 (1.0)	*2.1*	0 (0.0)	*0.0*	3 (3.4)	*7.5*
Blue-collar worker	323 (6.0)	*29.5*	131 (9.6)	*30.0*	29 (5.7)	*29.9*	25 (8.3)	*43.6*	48 (10.3)	*28.8*	24 (12.4)	*32.5*	6 (11.5)	*43.7*	12 (13.8)	*30.4*
Administrative staff	489 (9.0)	*12.1*	174 (12.7)	*11.5*	50 (9.9)	*13.9*	26 (8.7)	*11.5*	70 (14.9)	*11.7*	31 (16.1)	*11.8*	6 (11.5)	*10.1*	14 (16.1)	*12.1*
Executive staff	1,247 (23.0)	*10.2*	381 (27.9)	*9.4*	125 (24.6)	*10.2*	54 (18.0)	*6.7*	129 (27.5)	*8.4*	31 (16.1)	*4.2*	9 (17.3)	*6.2*	18 (20.7)	*5.6*
Retired	3,090 (57.1)	*16.6*	560 (40.9)	*9.5*	274 (53.9)	*14.4*	183 (61.0)	*16.2*	167 (35.7)	*7.5*	88 (45.6)	*7.5*	28 (53.9)	*11.4*	32 (36.8)	*5.2*
BMI, *kg/m* ^2^, mean, SD	25.2 (3.5)		24.8 (3.8)		25.3 (3.6)		28.3 (4.4)		25.4 (3.9)		28.2 (5.0)		29.0 (5.8)		28.4 (4.8)	
BMI categories																
Underweight <18.5	27 (0.5)	*1.4*	34 (2.5)	*3.2*	6 (1.2)	*1.4*	0 (0.0)	*0.0*	4 (0.9)	*3.8*	1 (0.5)	*0.2*	0 (0.0)	*0.0*	0 (0.0)	*0.0*
Normal weight 18.5–24.9	2,920 (53.9)	*60.7*	760 (55.6)	*54.9*	255 (50.2)	*53.4*	72 (24.0)	*32.9*	248 (53.0)	*47.1*	57 (29.6)	*14.5*	16 (30.8)	*26.1*	21 (24.2)	*10.3*
Overweight 25.0–29.9	1,990 (36.8)	*30.4*	448 (32.7)	*32.1*	198 (39.0)	*34.2*	137 (45.7)	*36.9*	169 (36.1)	*34.9*	79 (40.9)	*40.4*	17 (32.7)	*28.1*	39 (44.8)	*52.1*
Obesity ≥30.0	476 (8.8)	*7.5*	126 (9.2)	*9.8*	49 (9.6)	*11.0*	91 (30.3)	*30.2*	47 (10.0)	*14.2*	56 (29.0)	*44.9*	19 (36.5)	*45.8*	27 (31.0)	*37.6*
Smoking status																
Never smoker	2,197 (40.6)	*45.9*	622 (45.5)	*52.4*	171 (33.7)	*41.1*	102 (34.0)	*45.5*	189 (40.4)	*41.8*	70 (36.3)	*48.3*	13 (25.0)	*29.3*	28 (32.2)	*44.6*
Former smoker	2,744 (50.7)	*40.5*	611 (44.6)	*36.0*	289 (56.9)	*43.3*	175 (58.3)	*41.4*	217 (46.4)	*44.6*	105 (54.4)	*47.1*	35 (67.3)	*62.9*	49 (56.3)	*32.2*
Current smoker	472 (8.7)	*13.6*	135 (9.9)	*11.6*	48 (9.4)	*15.6*	23 (7.7)	*13.1*	62 (13.2)	*13.6*	18 (9.3)	*4.6*	4 (7.7)	*7.8*	10 (11.5)	*23.2*
Physical activity level^f^																
Low	923 (17.1)	*17.4*	318 (23.2)	*24.7*	86 (16.9)	*18.3*	68 (22.7)	*30.2*	123 (26.3)	*33.1*	52 (26.9)	*34.9*	17 (32.7)	*44.3*	16 (18.4)	*18.9*
Moderate	1,804 (33.3)	*35.4*	528 (38.6)	*39.0*	199 (39.2)	*38.3*	97 (32.3)	*36.4*	160 (34.2)	*29.2*	72 (37.3)	*30.7*	14 (26.9)	*36.8*	36 (41.4)	*33.3*
High	2,686 (49.6)	*47.2*	522 (38.2)	*36.3*	223 (43.9)	*43.4*	135 (45.0)	*33.4*	185 (39.5)	*37.7*	69 (35.8)	*34.4*	21 (40.4)	*18.9*	35 (40.2)	*47.8*

Values refer to number (percent) except when noted otherwise. Categorical variables compared across mental morbidity status using chi-squared tests; all p < 0.0001.

BMI, body mass index.

^a^ General anxiety proneness evaluated with STAI-T, applying sex-specific 75% cutoffs (men: 41 points; women: 46 points).

^b^ Chronic insomnia defined according to DSM-5, and ICSD-3, criteria.

^c^ Presence of any eating disorder defined as ≥2 affirmative responses on the SCOFF questionnaire.

^d^ W = percentage obtained by applying statistical weights according to sex-, age- and socioeconomic status distribution in the 2016 French Census.

^e^ The category includes individuals who are unemployed, homemakers, on sick leave, students, or interns.

^f^ Physical activity evaluated with the short form of the IPAQ, and scoring followed an established algorithm.

### Sociodemographic Profiles of Anxiety, Insomnia, and Eating Disorders Among Women

The largest proportion (45.9%) of women aged 18–39 years was found in the comorbid anxiety and ED group while the smallest proportion (10.5%) of women aged ≥60 years was found in the multimorbid anxiety-insomnia-ED group. The multimorbidity group also had the smallest proportions of women with executive positions or retired women (11.7% and 6.4%, respectively). Women with comorbid anxiety and ED had the highest prevalence of underweight (10.1%) and current smoking (18.8%) whereas women with insomnia and ED had the highest prevalence of overweight and obesity (31.9% and 23.0%, respectively).

### Sociodemographic Profiles of Anxiety, Insomnia, and Eating Disorders Among Men

Distinct sociodemographic profiles associated with mental morbidity in men were found (Table 3). The multimorbidity group had the smallest proportions of men aged ≥60 years (10.0%) and men without high school education (22.8%); in turn, it had the largest proportions of men who are overweight (52.1%) and current smokers (23.2%). Next, men with comorbid anxiety and ED were the most likely to be without professional activity (41.9%) and to live alone (60.7%) whereas men with comorbid insomnia and ED were the most likely to have obesity (45.8%) and low physical activity (44.3%).

## Discussion

According to WHO, mental disorders are widespread, undertreated, and under-resourced [6]. To help address this issue, this large, descriptive study focused on anxiety, insomnia, and ED owing to their relatively high prevalence and comorbidity in the general adult population, and the potential for prevention or treatment [10–12]. Among 33,658 individuals, a weighted total of 40.6% presented ≥1 mental disorder and an absolute total of 821 participants presented anxiety-insomnia-ED multimorbidity. Women were twice as likely as were men to have the latter (3.1% vs 1.4%, respectively). For most pure and comorbid conditions, women were at higher risk than were men.

Some distinct sex-specific sociodemographic profiles of mental morbidity emerged. Among women, having any ≥2 mental health conditions was associated with a higher prevalence of smoking than having ≤1 condition. Such a trend was absent among men. The largest proportions of younger adults (aged 18–39 years) were found among women with comorbid anxiety and ED and among men with pure anxiety, whereas the largest proportions of adults with obesity were found among those with comorbid insomnia and ED, across sex. Multimorbidity was associated with more risk behaviors among men than among women. The anxiety-insomnia-ED multimorbidity group had the largest proportions of men who were overweight (52.1%) and current smokers (23.2%), while no such trends emerged in women. These findings are not fully consistent with prior research in college students, showing that those with anxiety and/or mood disorders were more likely to be daily tobacco users compared to their counterparts without such mental disorders [40].

The present findings are largely consistent with prior mental health research reporting a high level of psychiatric comorbidity in the general population, observing that risk factor profiles for comorbid disorders differed considerably from those for pure disorders; such observations have important implications for prevention and clinical practice [17]. In addition, prior research regarding the 12-month comorbidity of anxiety, mood, and substance use disorders reported that the odds ratios for parental psychiatric history and childhood trauma were higher for comorbid anxiety-mood disorder, suggesting increased vulnerability and a more severe condition, than for either disorder in its pure form [17]. Also congruent with the present findings are previous reports of a positive association of mental comorbidity with female sex, living alone, and being of low socio-economic status [17–20]. Low physical activity and obesity have likewise been individually and positively associated with anxiety, ED, and insomnia/sleep disorders [41–45]. Even though ED types were not assessed in this study, the results regarding the prevalence of obesity suggest that restrictive types were unlikely to have driven the associations.

The frequent comorbidity of mental disorders, the shared risk factors and genetic expressions, and the observed activation in the same brain regions, especially those involved in emotion, learning and memory [7, 9, 46] have led some researchers to challenge the traditional view of mental disorders as distinct entities. To explain the underlying structure of mental illness, researchers have even proposed a general psychopathology continuum termed the “p factor” [47]. Likewise, the WHO stresses that mental health exists on a complex continuum [6]. Many physiological, genetic, affective, and cognitive mechanisms have been implicated in mental multimorbidity. For example, the association between anxiety and insomnia has been postulated to be driven by common gene variant heritability, dysregulation in circadian clock gene expression, serotonin, dopamine, and inflammatory cytokine secretion, and by cognitive inflexibility, interpretational biases, rumination tendencies, and impaired social interactions [48–50]. In addition, the role of the microbiome and the gut-brain axis in mental health have been attracting attention due to their involvement in numerous neuroendocrine, immune, inflammatory, and neurotransmitter pathways [51]. The bidirectional gut-brain communication is mediated by neural and humoral mechanisms. Whereas much work remains to be done in this area, it is known that intestinal dysbiosis and behavioral impairment are bidirectionally linked [51].

This descriptive study provided weighted prevalence estimates which argues for the external validity of the findings and against any selection bias. Specifically, the weighting largely compensated for differences (e.g., age, sex, educational level) between the study participants and the general French population. However, the cross-sectional design precludes any inference of causality. Likewise, the chronology of the three mental health conditions was beyond the scope of the study. There is evidence of complex bidirectional and possibly mediated associations among anxiety, insomnia, and ED [52–55]. Future longitudinal research could shed light on all of these issues. Another limitation, common to epidemiological research, pertains to the use of self-reported data provided by volunteers. We relied on DSM-5 and ICSD-3 criteria for insomnia, and on validated tools for anxiety (STAI-T) and ED (SCOFF); however, data obtained with these tools cannot serve as evidence for clinical diagnoses. A limitation of SCOFF is the lack of distinction among the various ED types. It should also be acknowledged that racial/ethnic background is an important component of the sociodemographic profile, however, such information was not available in our database.

In conclusion, this weighted analysis provided information about the prevalence and degree of mental multimorbidity of anxiety, insomnia, and ED among adults in the general population. Some distinct sex-specific sociodemographic profiles of mental morbidity emerged, which could be taken into consideration by targeted prevention programs, future cross-sectional and longitudinal mental multimorbidity research, and could help generate novel moderation and mediation hypotheses. In addition, the prevalence of mental comorbidity and multimorbidity justifies future methodological work aimed at generating mental multimorbidity composite measures. The findings also support the adaptation of medical education, public health guidelines, and healthcare services for multimorbidity [56, 57]. The advent of artificial intelligence in mental health will further necessitate the elaboration of novel regulatory frameworks, guidelines, and policies [58].
